# Analysis of the Antibiotic-Potentiating Activity, Absorption, Distribution, Metabolism, and Excretion (ADME) and the Molecular Docking Properties of Phytol Against Multi-Drug-Resistant (MDR) Strains

**DOI:** 10.3390/antibiotics13121171

**Published:** 2024-12-04

**Authors:** José Weverton Almeida-Bezerra, Saulo Almeida Menezes, José Thyálisson da Costa Silva, Simone Galdino de Sousa, Daniel Sampaio Alves, Gabriel Gonçalves Alencar, Isaac Moura Araújo, Ewerton Yago de Sousa Rodrigues, Cícera Datiane de Morais Oliveira-Tintino, Rafael Pereira da Cruz, Janaína Esmeraldo Rocha, Saulo Relison Tintino, José Maria Barbosa-Filho, Maria Flaviana Bezerra Morais-Braga, Irwin Rose Alencar de Menezes, António Raposo, Henrique Douglas Melo Coutinho

**Affiliations:** 1 Department of Biological Chemistry, Regional University of Cariri—URCA, Crato 63105-000, CE, Brazil; weverton.almeida@urca.br (J.W.A.-B.); isaac.moura@urca.br (I.M.A.); datianemorais@hotmail.com (C.D.d.M.O.-T.); irwin.alencar@urca.br (I.R.A.d.M.); 2 Biotechnology Center, Federal University of Rio Grande do Sul—UFRGS, Porto Alegre 91501-970, RS, Brazil; saulomenezes99@gmail.com; 3 Department of Biological Sciences, Regional University of Cariri—URCA, Crato 63105-000, CE, Brazil; jose.thyalisson@urca.br (J.T.d.C.S.); simonegaldino387@gmail.com (S.G.d.S.); danielsampaio9010@gmail.com (D.S.A.); gabriel.goncalves101@urca.br (G.G.A.); ewerton.sousarodrigues@urca.br (E.Y.d.S.R.); rafaelcruz284@gmail.com (R.P.d.C.); saulorelison@gmail.com (S.R.T.); flavianamoraisb@yahoo.com.br (M.F.B.M.-B.); 4 Center of Science and Technology CCT, State University of Ceara—UECE, Fortaleza 63100-000, CE, Brazil; janainaesmeraldo@gmail.com; 5 Department of Pharmacy, Federal University of Paraíba—UFPB, João Pessoa 58059-900, PB, Brazil; barbosa.ufpb@gmail.com; 6 CBIOS (Research Center for Biosciences and Health Technologies), Universidade Lusófona de Humanidades e Tecnologias, Campo Grande 376, 1749-024 Lisboa, Portugal

**Keywords:** antibacterial, antimicrobial resistance, diterpene, *Escherichia coli*

## Abstract

**Background:** Phytol is a diterpene from the long-chain unsaturated acyclic alcohols, known for its diverse biological effects, including antimicrobial and anti-inflammatory activities. Present in essential oils, phytol is a promising candidate for various applications in the pharmaceutical and biotechnological sectors. This study aimed to evaluate the *in vitro* antibacterial and drug-potentiating effects of phytol against multidrug-resistant bacteria and to evaluate its *in silico* properties: ADME and molecular docking. **Methods:** The *in vitro* antibacterial activity of phytol and the phytol combined with conventional drugs was evaluated by microdilution tests against standard and resistant bacterial strains. Finally, the SwissADME platform was employed to analyse the physicochemical and pharmacokinetic characteristics of phytol. **Results:** Phytol significantly reduced the Minimum Inhibitory Concentration (MIC) of norfloxacin and gentamicin required to inhibit multidrug-resistant strains of *Escherichia coli* and *Staphylococcus aureus*, respectively. Additionally, ADME analysis revealed that phytol exhibits low toxicity and favourable pharmacokinetic properties; in addition, it is revealed through molecular docking that phytol showed a relevant affinity with the proteins 6GJ1 and 5KDR, however, with values lower than the drugs gentamicin and ampicillin. **Conclusions:** Collectively, these findings suggest that phytol holds potential as an effective adjuvant in combating antimicrobial resistance.

## 1. Introduction

The use of antibiotics for treating infections began in the 1940s and led to a drastic reduction in deaths caused by bacterial infections [1,2]. However, unrestricted access to these drugs has resulted in indiscriminate use that leads to the selection of resistant microorganisms. Antimicrobial resistance has become one of the most serious public health concerns worldwide, with epidemiological studies showing that approximately 700,000 deaths occur annually due to microbial resistance [3,4].

Among resistant microorganisms, notable examples include the Gram-negative bacilli *Escherichia coli* (Enterobacteriaceae) and the Gram-positive cocci *Staphylococcus aureus* (Staphylococcaceae) [5]. The former group is characterised by its direct association with hard-to-treat infections and symptoms such as watery and bloody diarrhoea [6]. In contrast, *S. aureus* is responsible for a wide range of infections, affecting superficial skin and soft tissues, and even causing severe and potentially fatal systemic infections, such as endocarditis, pneumonia, and septicaemia [7,8].

Given these considerations, reversing resistance through the search for biologically active compounds is of paramount importance. Among the potential candidates are products derived from plant secondary metabolism, such as terpenes. These compounds stand out as a prominent option in antibacterial research due to their lipophilic characteristics, enabling them to interact with the components of bacterial cell membranes [9,10].

A significant member of this group is the diterpene phytol (C_20_H_40_O), characterised as a branched-chain unsaturated terpene, which exhibits antinociceptive, antioxidant, anti-inflammatory, antiallergic, and antibacterial activities. Additionally, this diterpene acts as an immunostimulant, making it a promising candidate for various applications in the pharmaceutical and biotechnological industries [11,12,13]. Therefore, due to phytol’s lipophilic capability to cross cell membranes [14], it is hypothesised to possess antibacterial activity and drug-enhancing potential.

However, for phytol to be effective as a medication, it must reach its target in the body at a concentration and for a duration sufficient to exert its antibacterial activity. Consequently, the development of new drugs necessitates the evaluation of pharmacokinetic properties, such as absorption, distribution, metabolism, and excretion (ADME), using computational models [15,16].

Considering the rise in bacterial drug resistance and the search for compounds with antibacterial properties, this study aimed to evaluate the *in vitro* antibacterial and drug-enhancing activity of the diterpene phytol against multidrug-resistant (MDR) bacteria and to investigate its ADME properties *in silico*.

## 2. Results and Discussion

### 2.1. Antibacterial and Drug-Potentiating Activity

When evaluating the antimicrobial activity of phytol against standard and MDR bacterial strains, it was observed that phytol did not exhibit direct antibacterial activity at clinically relevant concentrations (MIC > 512 μg/mL). Although previous studies have suggested that oxygenated terpenes display superior antibacterial activities compared to terpene hydrocarbons [10], our study demonstrates that the oxygenated diterpene phytol does not show direct antibacterial activity at clinically relevant concentrations [17]. This finding led us to discard the hypothesis that the diterpene, due to its chemical structure, would be a compound with significant antibacterial activity. Consequently, this contributes to understanding the limitations of the antimicrobial activities attributed to oxygenated terpenes.

Most research evaluating the antibacterial activities of plant secondary metabolites, such as essential oils, leaves a gap in the literature [10], as they often fail to attribute antimicrobial activities to major compounds (>20%) or synergistic effects between compounds. For instance, ref. [18] demonstrated that the essential oil of *Cleome spinosa* Jacq. (Cleomaceae), with phytol (31.3%) as a major constituent, exhibited antibacterial activity in disc diffusion tests against *Escherichia coli* (inhibition zone: 10 mm) and *Staphylococcus aureus* (inhibition zone: 12 mm). However, our study shows that the diterpene phytol does not exhibit direct antibacterial activity at clinically relevant concentrations in *in vitro tests*, suggesting that the activity of the essential oil may be responsible for synergistic action by the compounds, and not attributed only to phytol.

**Figure 1:**
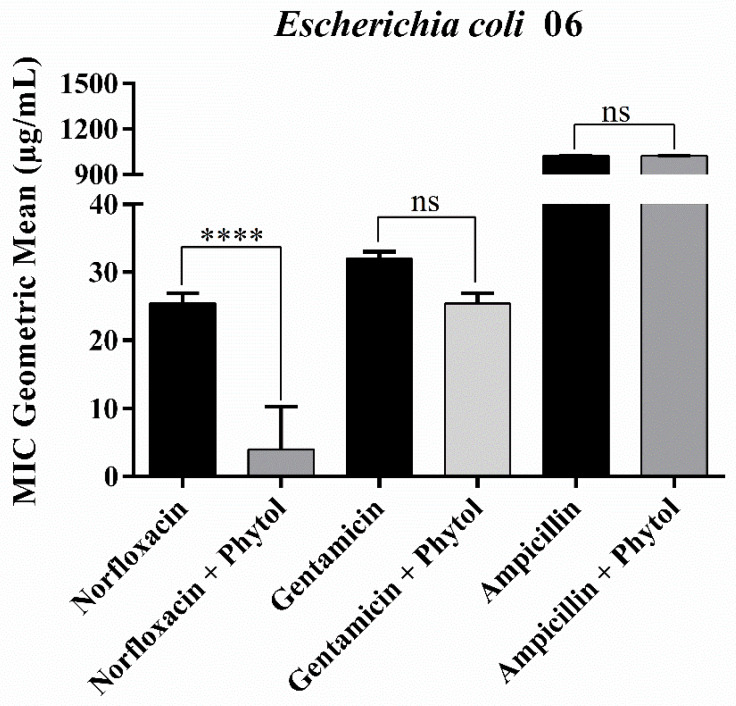
Minimum Inhibitory Concentration (MIC) of phytol in combination with antibiotics against the multidrug-resistant *Escherichia coli* 06 strain. ns: not significant (*p* > 0.05), ****: *p* < 0.0001. Bars represent the standard deviation of the geometric mean of MIC (*n* = 3).

Despite its lack of direct antibacterial activity, the diterpene was able to enhance the activity of norfloxacin, reducing its MIC from 25.39 µg/mL to 4 µg/mL and overcoming microbial resistance in *E. coli* 06. However, when combined with gentamicin and ampicillin, phytol did not enhance their effects (Figure 1). The negative control, used as a growth control, showed results consistent with the expected pattern of normal growth.

Some phytochemical compounds are inactive when used alone and only exhibit significant activity when administered in conjunction with an antibiotic [19]. This was observed with the *E. coli* 06 strain, which regained sensitivity when treated with a combination of phytol and norfloxacin. Similarly, *S. aureus* showed enhanced susceptibility to gentamicin when combined with phytol. Among the primary mechanisms by which phytochemicals enhance drug activity are the inhibition of efflux pumps, inhibition of beta-lactamase enzymes, and membrane permeabilisation. In the case of phytol, it may have increased membrane permeability, thereby facilitating the entry of norfloxacin [20].

**Figure 2:**
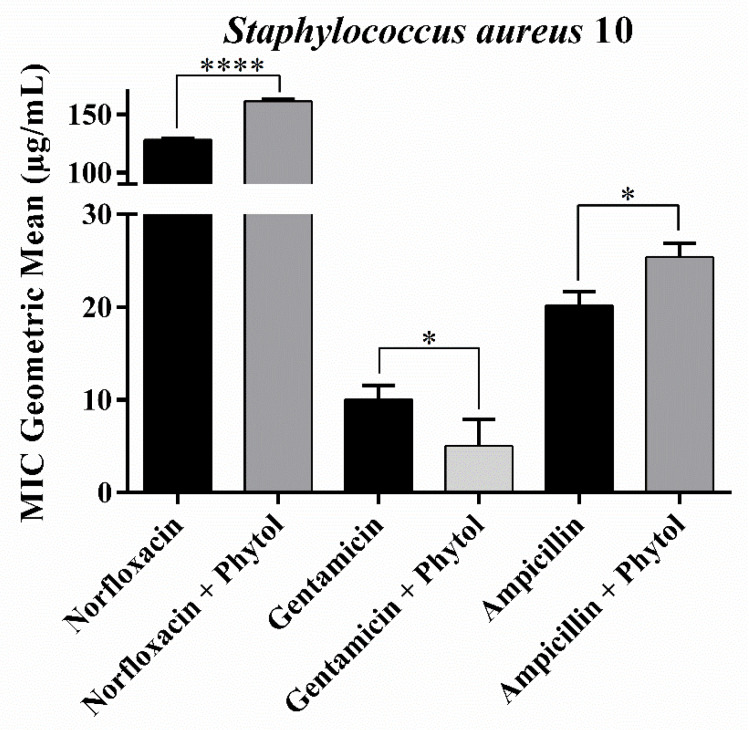
Minimum Inhibitory Concentration (MIC) of phytol in combination with antibiotics against the multidrug-resistant *Staphylococcus aureus* 10 strain. *: *p* < 0.01, ****: *p* < 0.0001. Bars represent the standard deviation of the geometric mean of MIC (*n* = 3).

However, when combined with norfloxacin and ampicillin, phytol exhibited an antagonistic effect reducing the activity of these drugs against *S. aureus* 10. In contrast, when combined with gentamicin, phytol enhanced the efficacy of the drug, decreasing the MIC by 50% (Figure 2). Silva [21] also demonstrated that the monoterpene eugenol (C_10_H_12_O_2_) demonstrated antagonistic activity to the antibacterial action of norfloxacin against *S. aureus* 10 in *in vitro* tests. The factors causing potential antagonism in antibiotic combinations remain poorly understood. Nonetheless, competition for the same microbial target and molecular interactions are the primary reasons for the development of antagonism [22].

Different studies highlight the antibacterial properties of phytol isolate, showing significant activity against Gram-positive and Gram-negative microorganisms [23,24]. In addition, its potential to inhibit bacterial virulence and as an antibiofilm agent is observed [25]. Tests performed against *S. aureus* (MIC of 31.25 μg/mL) and *Pseudomonas aeruginosa* (MIC of 62.5 μg/mL) indicate its ability to act as an antibacterial agent, which may interfere with the activity of bacterial dehydrogenase [26].

Diterpenes such as geranylgeraniol and teprenone showed bactericidal activity comparable to phytol in tests carried out with strains of *S. aureus*, as demonstrated in the work of [27]. Antimicrobial activity and low toxicity of phytol have also been observed in Gram-negative strains, such as *Escherichia coli*, with an MIC50 of 62.5 μg/mL [23]. In addition, phytol has been identified as the main bioactive agent in the methanol extracts of the leaves of *Adhatoda vasica* Nees. (Acanthaceae), showing activity against *Bacillus licheniformis* [13].

### 2.2. In Silico ADME Prediction

**Figure 3:**
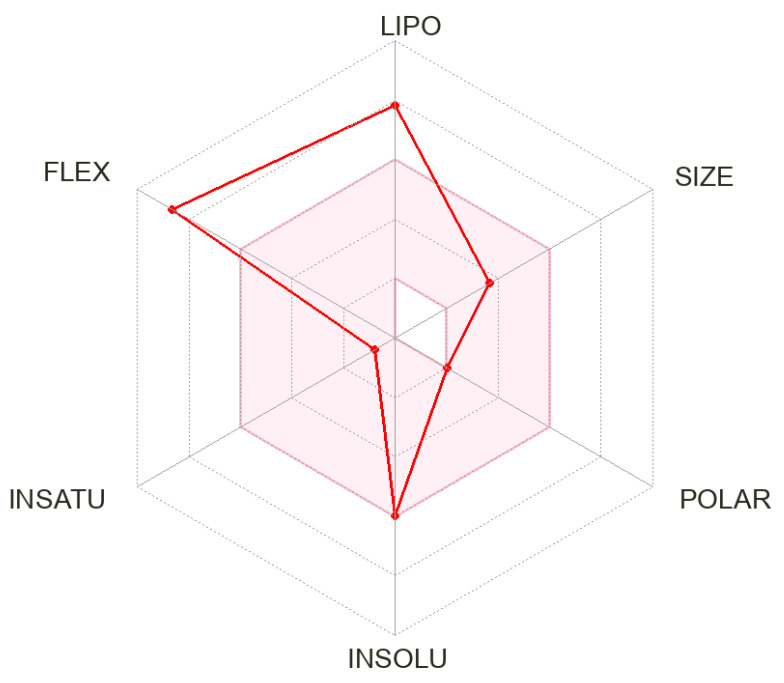
Bioavailability radar of phytol’s physicochemical properties compared to reference values of pharmaceutical medicines. The red line refers to the properties of Phytol.

According to the *in silico* analysis of phytol, the bioavailability radar of the compound’s physicochemical properties (Figure 3) illustrates the standard drug characteristics (shaded area) compared to those of phytol (red line). It is evident that the compound deviates from the properties favourable to drugs, failing to meet the criteria for lipophilicity (LIPO) and flexibility (FLEX). However, phytol meets all other drug-like properties, such as molecular size, polarity, solubility, and unsaturation, which may suggest some similarity to pharmaceuticals.

**Figure 4:**
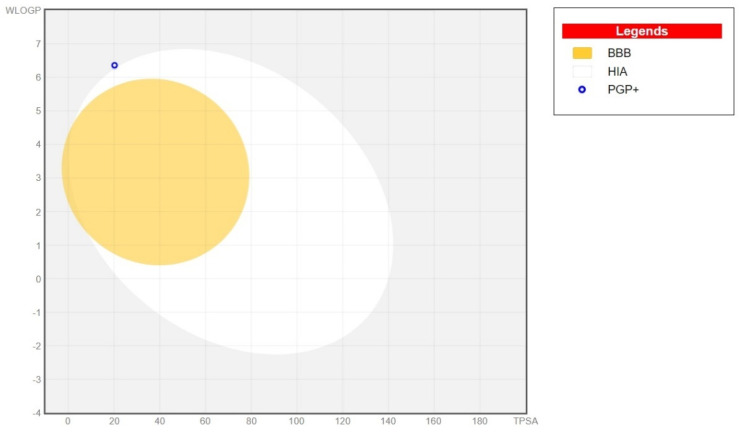
BOILED-egg graph representing the distribution and absorption properties of Phytol; HIA: human intestinal absorption; BBB: blood-brain barrier; P-gp; P-glycoprotein.

The BOILED-egg graph (Figure 4) illustrates the distribution and absorption of the compound within the body. Phytol has a low capacity to cross the blood–brain barrier (BBB), which may be advantageous in preventing potential neurotoxic effects. However, phytol also exhibits low human intestinal absorption (HIA), which could hinder its overall absorption. Additionally, the compound is a substrate of P-glycoprotein (Pgp), which is responsible for the active efflux of compounds.

The relevant physicochemical properties of phytol (Figure 3 and Figure 4) are summarised in Table 1. Aspects of flexibility, lipophilicity, and solubility are indicated as NRB (reference value: ≤9), XLOGP3 (reference value: −0.7 to +5.0), and log S (reference value: ≤6), respectively. The pharmacokinetic profile shows positive results, as there is no inhibition of the CYP isoenzyme complex, except for CYP2C9, which is essential for drug metabolism, and a moderate skin penetration (Log K) indicating potential for topical applications. It is also noted that phytol’s druglikeness and medicinal chemistry do not align with the rules of Ghose, Veber, Egan, and Muegge, particularly regarding the recommended lipophilicity (LOGP). Nonetheless, despite discrepancies in lipophilicity, Lipinski’s rules may still be applicable to phytol.

Pharmacokinetic and physicochemical characteristics are crucial parameters in studying substances with potential pharmacological activity, and computational *in silico* analysis generally facilitates this for a broader audience. These analyses contribute to predicting possible toxic activities, druglikeness, and molecular interactions [15,28,29]. Parameters such as Lipinski’s “Rule of 5”, which is based on the physicochemical properties of promising substances (MW ≤ 500, log P ≤ 5, NHD ≤ 5, NHA ≤ 10), are valuable indicators for active compounds. These parameters consider aspects such as solubility (Log P) and intestinal permeability (HIA), assisting in evaluating oral absorption and bioavailability [30,31].

The analysis of phytol’s intrinsic characteristics highlights positive aspects, such as its low toxicity and the absence of carcinogenic properties. Additionally, the substance complies with Lipinski’s rules, demonstrating good pharmacokinetic activity according to previous research [32,33]. *In vivo* studies conducted on Wistar rats confirmed these safety aspects, suggesting phytol’s potential as a multifaceted neuroprotective agent. These studies indicated a reduction in the expression of proteins associated with cell death [34] and mitigation of brain damage caused by parasites [35].

The results obtained reveal that phytol enhances the action of antibiotics such as norfloxacin. Supporting this finding, phytol was shown to have antibacterial activity against *Pseudomonas aeruginosa* (MIC = 20 μg/mL), indicating that its mechanism of action is similar to that of norfloxacin [36], which inhibits DNA replication [37]. There is growing interest in identifying compounds that enhance the efficacy of antibiotics in MDR bacteria by inhibiting resistance mechanisms such as efflux pumps [38].

The interaction of phytol with permeability glycoproteins (P-gp) and cytochrome P450 (CYP) isoenzymes is a significant aspect, as it can enhance the absorption and limit the metabolism of other compounds [39]. Our studies support this interaction, with evidence showing that phytol effectively inhibits the P-gp-mediated efflux pump by binding to the PAD site and acting against MDR pathogens [40]. This action is attributed to phytol’s ability to interfere with the NF-κB pathway, which is associated with inflammatory responses [41].

It is essential to investigate the toxicological aspects of promising compounds for human use as antibiotics. Phytol, in particular, exhibits a safe profile with low toxicity [13,32] and effectively combats oxidative damage by acting as an antioxidant through Nrf2 regulation [42,43]. However, it may cause adverse effects, such as hepatocellular necrosis in mice [44]. It is important to consider the dosage used, as toxic or cytotoxic activity is dose-dependent [45,46].

### 2.3. Molecular Docking

The analysis revealed that phytol exhibited similar binding affinities for both proteins, with values of −5.061 kcal/mol for 6GJ1 and −5.509 kcal/mol for 5KDR. In contrast, the antibiotics demonstrated higher binding affinities with these target proteins during molecular docking. For 5KDR (carboxyltransferase, *S. aureus*), ampicillin showed the highest affinity, with a value of −7.999 kcal/mol, whereas for 6GJ1 (T6SS, *E. coli*), gentamicin stood out with a binding affinity of −9.688 kcal/mol (Table 2).

**Figure 5:**
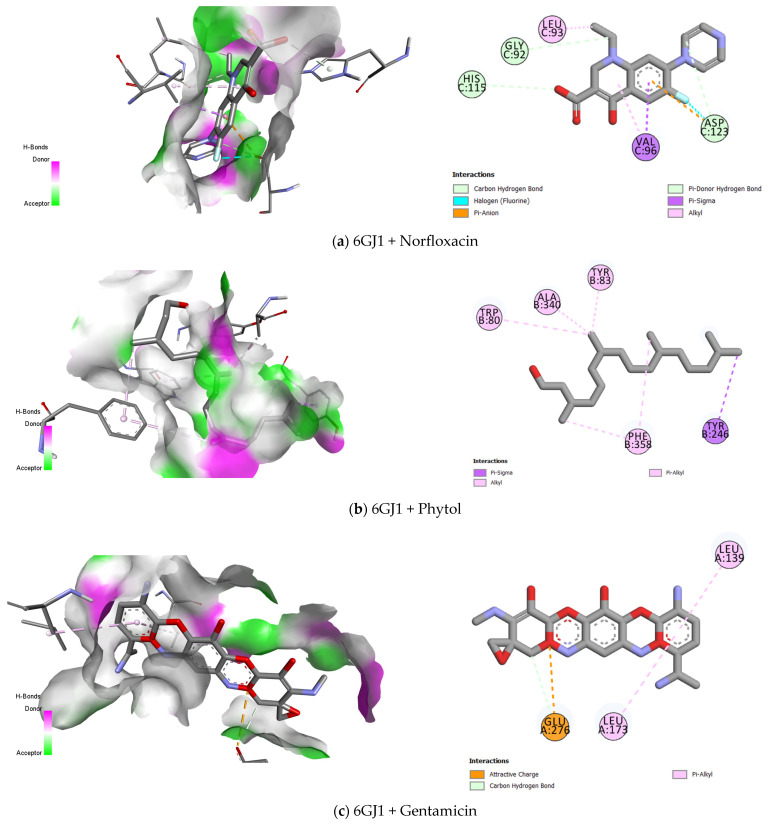

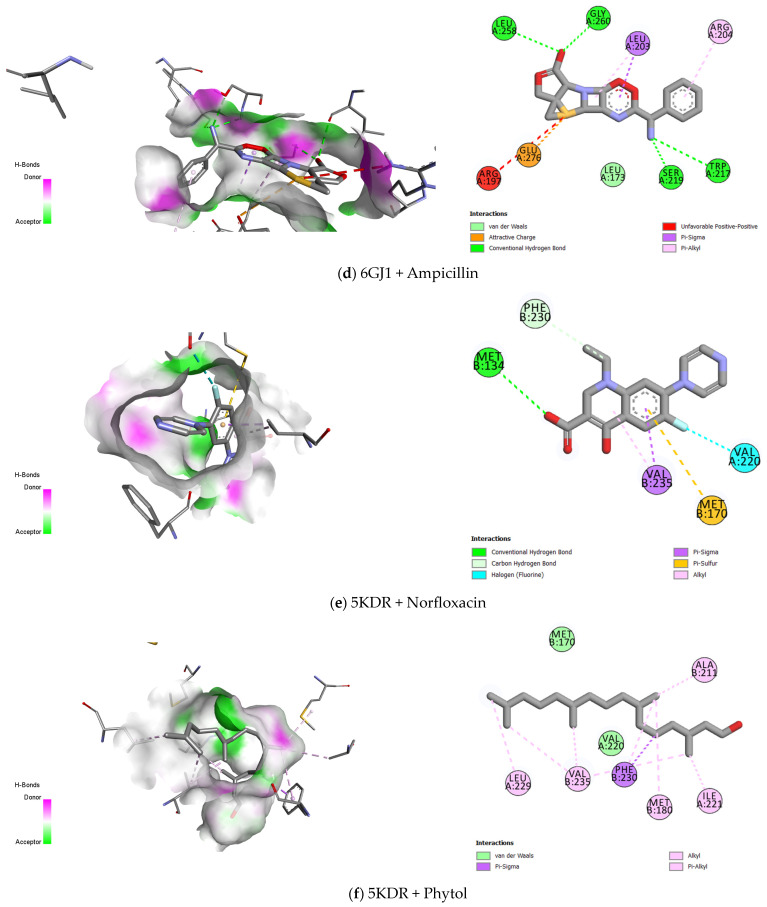

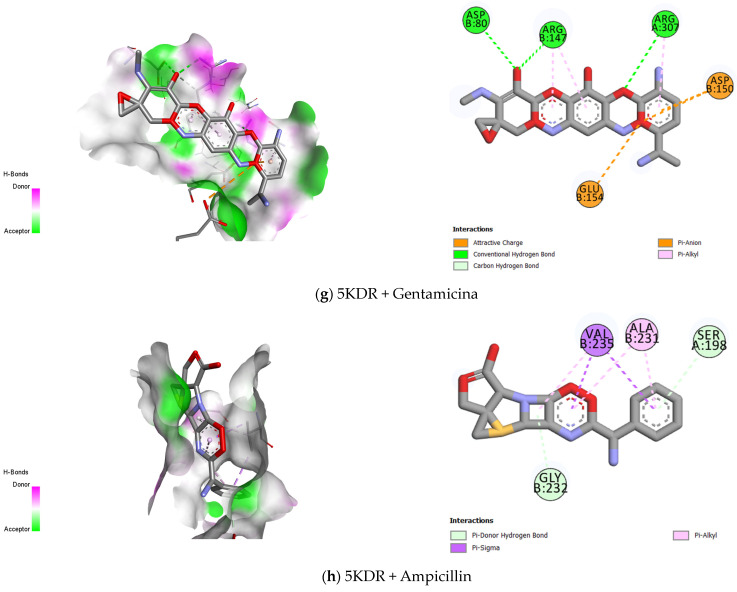
Description of the active sites and drug–receptor interactions between drugs (norfloxacin, phytol, gentamicin, and ampicillin) and proteins (5KDR and 6GJ1).

In the molecular docking analysis, phytol exhibited an affinity of −5.061 kcal/mol for the 6GJ1 protein, interacting with the binding sites through Pi-Sigma interactions (TYR B:246), Pi-Alkyl, and Alkyl interactions in the TRP B:80, ALA B:340, and TYR B:83 regions (Figure 5b). Regarding the 5KDR protein, the affinity was −5.509 kcal/mol, with van der Waals interactions (VAL A:220), Pi-Sigma interactions (PHE B:230), and Alkyl and Pi-Alkyl bonds involving the residues LEU A:229, VAL B:235, MET B:180, ALA B:211, and ILE A:221 (Figure 5f).

The carboxyltransferase enzyme is being investigated due to its relevance to the functionality of bacterial cells, making it a target protein for the development of antibacterial agents [47,48]. This enzyme, an essential component of acetyl-CoA carboxylase, plays a crucial role in the initial stages of fatty acid synthesis, which are fundamental for cell growth [49]. Thus, inhibition or alterations that compromise its functionality are considered promising strategies to combat pathogenic bacteria by interfering with their catalytic capacity [50,51]. Notably, natural product-derived substances, such as Moiramide B, have shown potential activity against this protein in *S. aureus* strains [52].

The antagonism observed in Figure 2, resulting from the combination of phytol with the drug norfloxacin in *S. aureus* strains, may be directly related to the action sites of the carboxyltransferase enzyme protein (Figure 5e,f). It is noted that both compounds share several binding sites, such as PHE B:230, MET B:170, VAL B:235, and VAL A:220, suggesting the possibility of competition for these same sites, which could explain the antagonistic interaction observed between the compounds [22].

In contrast to the antagonism identified (Figure 2), the combination of phytol and norfloxacin shown in Figure 1 demonstrates clinically relevant synergistic activity against *Escherichia coli*. This effect may be explained by the difference in the interaction sites of both compounds on the 6GJ1 protein (Figure 5a,b), as they act on distinct amino acids, thereby eliminating competition for binding sites. The 6GJ1 protein is associated with the Type VI secretion system (T6SS), a component of the protein machinery responsible for the direct transport of proteins to target cells, playing a significant role in virulence [53].

The Type VI secretion system (T6SS) is a protein structure of great importance for bacteria, both in growth within competitive environments and in pathogenesis. It operates by injecting specific toxins and is anchored to the plasma membrane complex [54]. Studies indicate that its synthesis, assembly, contraction, or disassembly does not impose significant costs on bacterial cells [55]. This functionality directly influences the virulence, resistance, and protection of bacteria such as *Escherichia coli*, contributing to the high prevalence and increasing rates of intestinal and extraintestinal infections caused by this species [56,57].

The T6SS is considered a strategic target for the development of new therapeutic antimicrobial agents, with the potential to aid in combating pathogenic bacteria and those resistant to conventional drugs. This structure plays a crucial role in the formation, establishment, stability, and evolution of the microbiota [58,59]. Furthermore, the absence of a functional T6SS has been associated with negative impacts on the evolution and success of multidrug-resistant *E. coli* clones [60].

## 3. Materials and Methods

### 3.1. Antibiotics and Reagents

For the *in vitro* antibacterial assays, norfloxacin (fluoroquinolone), gentamicin (aminoglycoside), and ampicillin (penicillin) from Sigma (Co., St. Louis, MO, USA) were used. These antibacterial drugs were diluted in sterilised distilled water to a concentration of 1.024 μg/mL. The diterpene phytol (Sigma-Aldrich^®^) was initially diluted in dimethyl sulfoxide (DMSO) and, subsequently, in sterilised distilled water to reach a concentration of 1.024 μg/mL, with a final DMSO concentration of 10%. The antibacterial assays were read using sodium resazurin (Sigma-Aldrich, St. Louis, MO, USA).

### 3.2. Culture Media and Bacterial Strains

**Figure 6:**
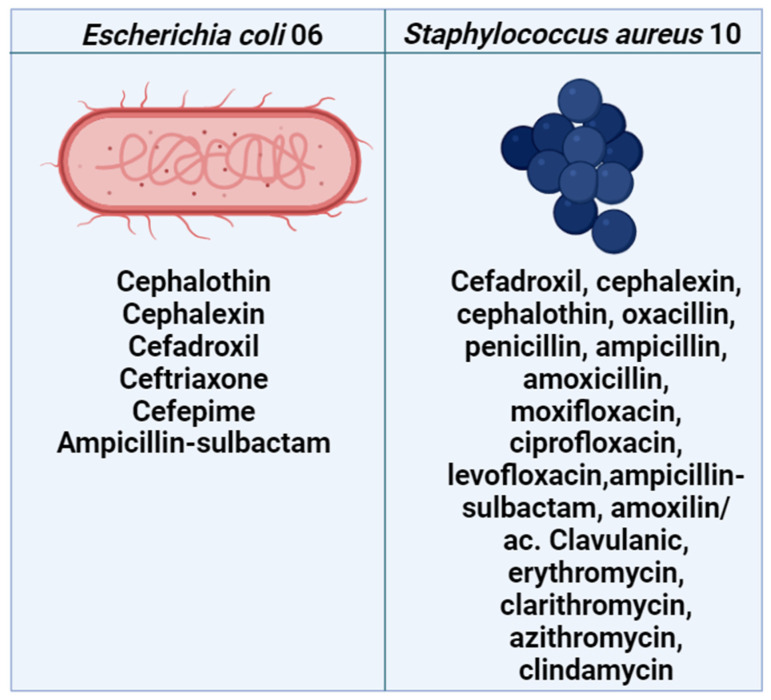
Resistance profile of *Escherichia coli* 06 and *Staphylococcus aureus* 10. Source: Laboratory of Microbiology and Molecular Biology (LMBM), Regional University of Cariri (URCA).

Brain Heart Infusion (BHI) broth (Kasvi) was prepared at a 10% concentration for the microdilution assays. What is composed of: HM Infusion powder: 12.50 g/L, BHI Powder: 5.00 g/L, Proteose peptone: 10.00 g/L, Dextrose (Glucose): 2.00 g/L, Sodium chloride: 5.00 g/L, Disodium hydrogen phosphate: 2.50 g/L, Final pH (at 25 °C): 7.4 ± 0.2. Mueller–Hinton Agar (Kasvi) was used for bacterial strain growth and prepared according to the manufacturer’s instructions. It is composed of the following: peptone, 17.9 g; BHI, 5 g; infusion solids, 6.25 g; starch, 15 g; glucose, 20 g; and final pH, 7.5 ± 0.2. Standard strains (*Escherichia coli* ATCC 25922 and *Staphylococcus aureus* ATCC 25923) and MDR strains (*Escherichia coli* 06 and *Staphylococcus aureus* 10) were used. The MDR *E. coli* and *S. aureus* strains originated from urine and rectal swab cultures, respectively, with resistance profiles described in Figure 6. All bacteria were stored at 4 °C before *in vitro* assays and were cultured on Mueller–Hinton Agar prior to testing. They were then incubated in an incubator at 37 °C for 24 h.

### 3.3. In Vitro Antibacterial Activity

The Clinical and Laboratory Standards Institute [61] serial microdilution method in broth was used to determine the Minimum Inhibitory Concentration (MIC). Bacterial strains were suspended in a test tube containing sterile saline (0.9%) using a nickel–chrome inoculation loop until a bacterial load of 10^5^ CFU was achieved according to the McFarland scale.

Subsequently, 96-well plates (Kasvi) were filled with 100 µL of BHI broth containing a 10% bacterial inoculum. Serial dilutions (1:1 *v*/*v*) of phytol were then performed up to the penultimate well (designated as the growth control), achieving concentrations ranging from 4 to 512 μg/mL. Sterility control plates were prepared with only BHI broth and sterile saline. The plates were incubated at 37 °C for 24 h. After incubation, 20 µL of sodium resazurin solution was added to each well, and readings were taken after one hour. The MIC was defined as the lowest concentration that inhibited microbial growth.

### 3.4. Drug-Potentiating Activity

In addition to assessing the intrinsic antibacterial activity, the potential drug-enhancing activity was evaluated. For this purpose, phytol was tested at sub-inhibitory concentrations (MIC/8) in combination with the antibiotics norfloxacin, gentamicin, and ampicillin [62]. The diterpene phytol (MIC/8) was added to BHI broth containing a 10% bacterial inoculum and then microdiluted (1:1 *v*/*v*) with the aforementioned antibiotics. This procedure followed the same protocol described in Section 3.3. Additionally, antibiotic controls were prepared, containing only BHI broth and bacterial inoculum, and serial microdilution (1:1 *v*/*v*) was performed.

### 3.5. In Silico ADME Prediction

To analyse the physicochemical and pharmacokinetic characteristics of phytol (C_20_H_40_O), the Canonical SMILES (Simplified Molecular Input Line Entry System) provided by PubChem (https://pubchem.ncbi.nlm.nih.gov/ (accessed on 19 November 2024)) was used. This was submitted to the SwissADME platform (http://www.swissadme.ch/ (accessed on 22 July 2024)), provided by the Swiss Institute of Bioinformatics (SIB), with a focus on toxicological parameters, BOILED-egg, and the bioavailability radar [15].

### 3.6. Statistical Analysis

The antibacterial assays were conducted in triplicate, with geometric means and their respective standard deviations calculated. Subsequently, the data were subjected to a one-way analysis of variance (ANOVA) followed by Tukey’s post hoc test. Results were considered significant when *p* < 0.05 and *p* < 0.0001, and not significant at *p* > 0.05. Statistical analyses were performed using GraphPad Prism version 5.0.

### 3.7. Molecular Docking

#### 3.7.1. Protein of Interest

The target proteins (PDB IDs: 5KDR and 6GJ1) and their respective ligands were obtained from the Protein Data Bank (PDB), a repository that stores data on proteins and their three-dimensional structures. Each PDB entry includes various types of information, such as atomic coordinates in three-dimensional space, polymer sequences, and metadata [63]. The removal of the protein inhibitor and water molecules from the receptor structure was performed using Discovery Studio 2021 client software (21.1.0.20298).

#### 3.7.2. Binder Treatment

The compounds phytol, gentamicin, norfloxacin, and ampicillin were selected for *in silico* evaluation by molecular docking. The ligands used in the study were modelled in 3D with ACD/ChemSketch software, while 2D models were obtained from ChemSpider (Cambridge, UK) for phytol (ID: 4444094), norfloxacin (ID: 4380), gentamicin (ID: 26354731), and ampicillin (ID: 6013). The “rigid-flexible protein ligand” docking process was conducted using the Autodock VINA system in PyRx software [64,65]. After docking, the most stable conformations of the ligands were analysed with Discovery software.

#### 3.7.3. Grid Calculation and Adjustment

The grid calculation was performed using 100 conformations in the Auto-dock VINA system of the PyRx software. For ligand–protein docking, the grid dimensions on the X, Y, and Z axes were set at 93.554 × 105.137 × 121.919 Å for 6GJ1 and 0.42 × 0.807 × 14.331 Å for 5KDR, with a spacing of 0.375 Å. The grid centre was set to 126, 126, and 126 Å in the case of the 6GJ1 protein and to 62, 62, and 62 Å in the case of 5KDR. The definition of the binding site was based on ligands previously cocrystallised with the proteins and available in the Protein Data Bank. The interaction energies between the ligands and the amino acid residues of the 5KDR and 6GJ1 proteins were determined using Discovery Studio software, which calculates the free binding energy considering components such as van der Waals, electrostatic interactions, and hydrogen bonds [66].

## 4. Conclusions

This study highlights the significant potential of phytol as an adjuvant in the fight against antimicrobial resistance. Our findings demonstrate that phytol enhances the efficacy of conventional antibiotics such as norfloxacin and gentamicin against multidrug-resistant strains of *Escherichia coli* and *Staphylococcus aureus*. Furthermore, the *in silico* ADME analysis indicates that phytol has favourable pharmacokinetic properties and low toxicity. Furthermore, molecular docking analysis revealed that phytol exhibits significant affinity for the proteins 6GJ1 and 5KDR, although with lower binding values compared to the drugs gentamicin and ampicillin, reinforcing its viability as a therapeutic agent. Given these promising results, phytol appears to be a valuable candidate for further development as a component in antimicrobial therapies, potentially offering a new strategy to overcome the challenges posed by resistant bacterial infections.

This study has restrictions about the pathways and mechanisms of action of phytol as an antibacterial agent, as well as the detailed analysis of its pharmacokinetics, highlighting the need for future investigations to analyse its pharmacological safety. In addition, it is essential to carry out *in vivo* tests, as well as to evaluate potential toxic effects, including cytotoxicity and genotoxicity.

## Figures and Tables

**Table 1:** Physicochemical, pharmacokinetics, druglikeness, and medicinal chemistry properties of phytol.

**Physicochemical Properties**	
MF	C_20_H_40_O
MW	296.53 g/mol
NRB	13
NHA	1
NHD	1
TPSA	20.23 Å^2^
Log *P*_o/w_ (XLOGP3)	8.19
Log *S* (ESOL)	−5.98
**Pharmacokinetics**	
HIA	Low
BBB	No
P-gp substrate	Yes
CYP1A2 inhibitor	No
CYP2C19 inhibitor	No
CYP2C9 inhibitor	Yes
CYP2D6 inhibitor	No
CYP3A4 inhibitor	No
Log *K*_p_ (cm/s)	−2.29 cm/s
**Druglikeness**	
Lipinski	Yes (1)
Ghose	No (1)
Veber	No (1)
Egan	No (1)
Muegge	No (2)
**Medicinal Chemistry**	
PAINS	0
Brenk	1: isolated_alkene
Leadlikeness	No (2)
Synthetic accessibility	4.30

MF: molecular formula; MW: molecular weight; NRB: number of rotatable bonds; NHA: number of hydrogen acceptors; NHD: number of hydrogen donors; TPSA: topological polar surface area; HIA: human intestinal absorption; BBB: blood–brain barrier; P-gp; P-glycoprotein; CYP: Cytochrome-P.

**Table 2:** Affinity of protein and compounds with different types of binding, 6GJ1, and 5KDR proteins.

Protein	Compounds	Biniding Affinites (kcal/mol)	Bond Type
6GJ1–*E. coli*	Norfloxacin	−7.673	Carbon–Hydrogen bond
			Halogen (Fluorine)
			Pi- Anion
			Pi- Donor Hydrogen Bond
			Pi- Sigma
			Alkyl
	Gentamicin	−9.688	Attractive charge
			Carbon–Hydrogen bond
			Pi- Alkyl
	Ampicillin	−9.105	van der Waals
			Attractive Charge
			Conventional Hydrogen Bond
			Unfavourable Positive–Positive
			Pi- Sigma
			Pi- Alkyl
	Phytol	−5.061	Pi- Sigma
			Pi- Alkyl
			Alkyl
5KDR–*S. aureus*	Norfloxacin	−6.687	Conventional Hydrogen Bond
			Carbon–Hydrogen bond
			Halogen (Fluorine)
			Pi- Sigma
			Pi- Sulphur
			Alkyl
	Gentamicin	−7.59	Attractive Charge
			Conventional Hydrogen Bond
			Carbon–Hydrogen bond
			Pi- Anion
			Pi- Alkyl
	Ampicillin	−7.999	Pi- Donor Hydrogen Bond
			Pi- Sigma
			Pi- Alkyl
	Phytol	−5.509	van der Waals
			Pi- Sigma
			Alkyl
			Pi- Alkyl

## Data Availability

Data are contained within the article.
